# Molecular Landscape of Acute Myeloid Leukemia: Prognostic and Therapeutic Implications

**DOI:** 10.1007/s11912-020-00918-7

**Published:** 2020-06-01

**Authors:** Ludovica Marando, Brian J. P. Huntly

**Affiliations:** 1 Wellcome Trust-MRC Cambridge Stem Cell Institute, Cambridge Biomedical Campus, Cambridge, UK; 2 Department of Haematology, University of Cambridge, Cambridge, UK

**Keywords:** Genomics, Mutations, Prognosis, Preleukemia, Clonal evolution, Targeted therapy

## Abstract

**Purpose of Review:**

The field of acute myeloid leukemia (AML) has been revolutionized in recent years by the advent of high-throughput techniques, such as next-generation sequencing. In this review, we will discuss some of the recently identified mutations that have defined a new molecular landscape in this disease, as well as their prognostic, predictive, and therapeutic implications.

**Recent Findings:**

Recent studies have shown how many cases of AML evolve from a premalignant period of latency characterized by the accumulation of several mutations and the emergence of one or multiple dominant clones. The pattern of co-occurring mutations and cytogenetic abnormalities at diagnosis defines risk and can determine therapeutic approaches to induce remission. Besides the genetic landscape at diagnosis, the continued presence of particular gene mutations during or after treatment carries prognostic information that should further influence strategies to maintain remission in the long term.

**Summary:**

The recent progress made in AML research is a seminal example of how basic science can translate into improving clinical practice. Our ability to characterize the genomic landscape of individual patients has not only improved our ability to diagnose and prognosticate but is also bringing the promise of precision medicine to fruition in the field.

## Introduction

Acute myeloid leukemia is an aggressive and highly heterogeneous disease, with biologically and prognostically different subtypes [1]. Over 40 years have passed since pioneering work by Janet Rowley defined AML as a genetic disease with the description of the t(15;17) as a recurrent event in acute promyelocytic leukemia (APML) [2, 3]. Over the last few decades, we have witnessed a growing application of several high-throughput sequencing technologies, including whole genome, exome, and panel-based capture sequencing, that have helped refine the classification and prognostic scoring of AML. Current state-of-the-art iterations of these tools, the WHO classification (2016) and the European Leukemia Net (ELN) guidelines, have become the “industry standards.”

Well-established poor-risk prognostic variables in AML include older age, secondary disease, and adverse cytogenetics; however, recent analyses have also suggested the value of incorporating gene mutations beyond *FLT3*, *NPM1*, and *CEBPA* (e.g., *IDH1*and *IDH2*, *ASXL1*, *MLL*, *DNMT3A*, and *TET2*) into AML risk classifications [4]. Despite the advances in our understanding of the pathogenesis of AML, the standard of care still remains based around a “one size fits all” approach of age-adapted remission induction with chemotherapy and post-remission consolidation with chemotherapy and/or allogeneic hematopoietic stem cell transplant in younger patients. However, as the mutational landscape of AML is mapped in more detail, our therapeutic expectation in AML must evolve to match our increasing understanding of AML pathogenesis, with potential associated therapeutic vulnerabilities identified.

## Cytogenetics in the DNA Era

Diagnostic karyotype remains the most powerful prognostic indicator in AML and forms the basis of current prognostic scores. To give an example of the key role cytogenetics plays in the diagnosis of AML, the WHO classification has established that patients with the clonal, recurring cytogenetic abnormalities t(8;21)(q22;q22), inv. (16)(p13q22) or t(16;16)(p13;q22), and t(15;17)(q22;q12) should be considered to have AML regardless of the blast percentage [5]. By dividing patients into three risk groups, favorable, intermediate, and adverse [6], cytogenetics has been widely adopted to provide the framework for risk-adapted treatment approaches [7]. In certain situations, cytogenetics also allow for prediction of effective therapy; in patients with the t(15;17)(q22;q21)/*PML-RARA*, for instance, the combination of all-*trans* retinoic acid (ATRA) and anthracycline-based protocols has resulted in a markedly improved outcome. In contrast patients with complex karyotype (≥ 3 or ≥ 5 abnormalities depending on the classification system), monosomal karyotype (such as monosomy 5/del(5q) or monosomy 7/del(7q)), or abnormalities of 3q have been shown to have inferior complete remission rates and overall survival and are currently considered for allogeneic stem cell transplant in first remission.

However, although cytogenetic analysis remains mandatory in the evaluation of suspected myeloid leukemia, it also presents several limitations. Apart from technical failures, cytogenetics cannot identify cryptic rearrangements, for example, 5% of PML/RARA positive AML lack the classic t (15, 17), with the fusion gene resulting from more complex rearrangements [8]. These patients not only respond to targeted therapy in a similar fashion to patients with the classic translocation but also share the same favorable prognosis and requirement for ATRA to prevent catastrophic coagulopathy, therefore cannot be missed. Moreover, around 40–50% of adult and 25% of pediatric AML patients have a normal karyotype (CN-AML), and these individuals are highly heterogeneous in terms of clinical outcomes [11]. Therefore, improving risk stratification and clinical decision making for this group of patients is a vibrant focus of research. In this effort, the mutational analysis of *FLT3*, *NPM1*, and *CEBPA* has become standard practice to improve their risk stratification [12]. There are however several additional gene mutations that also appear to carry prognostic relevance that include *IDH1*, *IDH2*, *KIT*, *WT1*, and *RUNX1* [13], and their incorporation into risk stratification scoring is a matter of ongoing debate.

## The Molecular Landscape of AML

The advent of massive parallel sequencing heralded a new age in molecular diagnosis, prognosis, and prediction. AML was the first cancer genome to be sequenced [14] and remains one of the most highly sequenced tumors, with ease of access of tumor tissue an obvious facilitating factor. However, even prior to this, a number of candidate gene studies had determined point and more complex mutations in critical genes, and we will summarize these below.

## The Nucleolar Protein Nucleophosmin 1 (*NPM1*)

Mutations in the *NPM1* gene are among the most common genetic changes in AML (occurring in 25–35% of patients), especially in CN-AML (present in 45–64%) [15]. NPM1 plays a role in numerous cellular functions, including ribosome biogenesis, DNA repair, and regulation of apoptosis. More than 40 different mutations in the C-terminal region of the protein have been described, but these uniformly result in the disruption of an N-terminal nucleolar localization signal and cause the protein to be aberrantly localized to the cytosol [16]. *NPM1* mutations appear to be late driver events often occurring after *DNMT3A*, *IDH1*, or *NRAS* mutations [9••]. Interestingly, however, *NPM1*-mutated AML behaves as an entity on its own and is the largest classification category in a recent 11 component classification [9••]. *NPM1* mutations are not normally observed in patients with AML associated recurrent translocations, and murine models of *NPM1* mutation are associated with expanded myelopoiesis and the development of AML [17]. The prognostic implications of *NPM1* mutations in individual patients are highly dependent on the pattern of co-occurring mutations and confer favorable prognosis only if associated with *FLT3-ITD* wild-type or low allelic ratio. Growing evidence suggests that carrying an NPM1 mutation confers sensitivity to novel agents such as venetoclax [10].

## Mutations in Signaling Pathway Components

### Fms-Like Tyrosine Kinase 3 (*FLT3*)

*FLT3* is a tyrosine kinase that acts as a cytokine receptor for the *FLT3* ligand. First described in 1991, *FLT3* is strongly expressed in hematopoietic stem cells with important roles in cell survival and proliferation [18]*. FLT3* mutations are among the most common mutations in AML and occur as either in-frame duplications within the juxtamembrane region (*FLT3-ITD*, internal tandem duplication) or as point mutations within the tyrosine kinase domain (*FLT3-TKD*) at a frequency of around 25% and 7% of AML cases, respectively [1]. Both mutations constitutively activate the tyrosine kinase leading to enhanced RAS, MAPK, and STAT5 signaling that results in blast proliferation [19, 20]. The effect on prognosis is modulated by the mutated to wild-type allele ratio. This may reflect a dominant clone and/or uniparental disomy of Ch13 on which FLT3 resides and an increased ratio is associated with an inferior outcome. In addition, *FLT3-ITD* mutations are associated with increased risk of relapse, whereas the prognostic relevance of *FLT3-TKD* mutations remains controversial [21]. Recent studies have suggested that inhibitors of FLT3 are effective as single agents in the relapsed refractory setting, as up-front adjuvants to conventional therapy in newly diagnosed patients and possibly in the maintenance setting also (RATIFY, QuANTUM-R and ADMIRAL studies).

With the ability to sequence AML genomes, it has become apparent that a number of other genes encoding for signaling pathways components (*RAS*, *cKIT*, *NF1*, and others) are mutated in AML. The observation that mutations in signaling pathways proteins frequently co-occur with chromosomal rearrangements in hematopoietic transcription factors (*PML-RARA*, *CBFβ-MYH11*, *RUNX1-RUNX1T1*) led to the hypothesis that AML results from the cooperation of mutations that confer a proliferative advantage (class I mutations) with mutations that induce a block in differentiation (class II mutations), the 2-hit model [1]. However, over 40% of AML cases lack mutations in classical signaling pathway genes, suggesting that the evolution of acute leukemia is a more complex and individual phenomenon [22].

## Mutations in Epigenetic/Chromatin Modifiers

In recent years, a number of epigenetic and chromatin modifiers have been identified as mutated in AML. These mutations are classically found at the highest variant allele frequencies (VAF) in AML patients [9••] and can also persist in remission, [23] leading to the acceptance that these mutations often represent preleukemic events [24]. In the most recent large classification of AML, this group was also demonstrated to have a poor prognosis, [9••] a finding recapitulated for individual genes [25]. Efforts are therefore ongoing to dissect this group further, as epigenetic and chromatin modifiers represent effective therapeutic targets [26].

### DNA Methyltransferase 3A (*DNMT3a*)

*DNMT3A* mutations occur in 18–22% of all AML cases and approximately 34% of CN-AML cases [27], and they are mostly heterozygous and commonly affect a hotspot encoding arginine at codon 882 (~ 60% of AML cases). R882 mutations appear to result in a hypomorphic protein that acts in a dominant negative manner, inhibiting the methyltransferase activity of the remaining wild-type *DNMT3A* [28]. In murine models, when *Dnmt3a* is conditionally deleted, self-renewal is favored over differentiation [29], but the underlying mechanisms, and their relationship to DNA methylation, remain unexplained. The prognostic significance of *DNMT3A* mutations remain controversial, but recent evidence suggests that *DNMT3A*-mutated AML patients may benefit from higher doses of anthracyclines [30].

### Ten–Eleven Translocation 2 (*TET2*)

*TET2* is found mutated in about 9%–23% of AML patients [31]. *TET2* regulates the initial step in DNA demethylation through the conversion of 5-methylcytosine (5mC) to 5-hydroxymethylcytosine (5hmC). In general, *TET2* mutations are thought to be loss-of-function mutations; however, despite several studies, their prognostic significance remains unclear.

### Additional Sex Comb-Like 1 (*ASXL1*)

*ASXL1* loss-of-function mutations occur in ~ 5–11% of AML cases [32]. Although their mechanisms of action are not fully known, it is likely that they function, at least in part, through a loss of PRC2 (polycomb repressive complex 2) function allowing derepression of genes such as *HOXA* cluster genes [33]. *ASXL1* mutations are five times more common in older (> 60 years) patients, are frequently associated with t(8;21), wild-type *NPM1*, wild-type *FLT3*, and mutated *CEBPA*, and are considered adverse prognostic factors according to European Leukemia Net (ELN) criteria.

### Isocitrate Dehydrogenase (*IDH*)

*IDH* 1 and 2 gene mutations are neomorphic gain-of-function mutations that cause an alteration of normal function, allowing the mutant enzyme the novel function of further converting α-ketoglutarate into 2-hydroxyglutarate (2-HG). This “oncometabolite” inhibits the function of *TET2* and other dioxygenase enzymes causing effects on DNA and histone methylation and thus epigenetic regulation [34]. *IDH1* mutations more commonly affect the highly conserved arginine (R) residue at codon 132 (R132) and have been identified in 7% of AML patients. *IDH2* mutations are identified in a further 9% of cases and cluster at codons R140 and R172 [1]. Interestingly *IDH2* mutations are prognostically distinct, whereas R140 mutations are often associated with *NPM1* and predict more favorable outcomes, and R172 mutations seem to represent a distinct genomic subgroup with mutual exclusivity with NPM1 and indicate poorer prognosis [9••].

## Cohesin Complex

Cohesin is a large ring-shaped multi-protein complex consisting of four major subunits: SMC1A, SMC3, RAD21, and STAG1/2. Cohesin plays a role not only in mediating sister chromatid cohesion during mitosis, where it coordinates ordered chromosome separation and prevents mitotic catastrophe, but is also involved in DNA damage repair and the regulation of gene expression through coordinating interaction between distal and proximal *cis*-regulatory elements. Mutations in Cohesin subunits affect around 10% of AML patient [35] and typically co-occur with *NPM1*, *DNMT3A*, *TET2*, or *RUNX1* mutations [36]. Cohesin mutations or knockdown of Cohesin subunits impair hematopoietic differentiation and enforce stem cell programs in both human and mouse hematopoiesis. Furthermore, studies have demonstrated alterations of chromatin accessibility upon depletion of Cohesin function and have further linked AML development with a requirement of Cohesin function for dynamic gene expression during erythroid differentiation and interaction with ETS transcription factors [37, 38].

## RNA Splicing Factor Mutations

A number of RNA splicing factors are mutated in AML. The most commonly mutated genes include *SF3B1*, *U2AF1*, *SRSF2*, and *ZRSR2* [39]. Often considered founding events, splicing factor gene mutations are frequently found in preleukemic conditions such as MDS. In newly diagnosed AML patients, spliceosome gene mutations are now considered pathognomonic of secondary AML developing from preceding MDS [40]. These mutations are likely to alter splicing and subsequent translation of critical genes and are generally associated with poorer responses.

## Transcription Factor Mutations

### Runt-Related Transcription Factor (*RUNX1*)

The *RUNX1* gene is a partner in the t(8;21) fusion gene in CBF leukemia and is also affected by recurrent gene mutations in AML [41]. *RUNX1* mutations are found in 5–13% of AML cases and are commonly associated with trisomy 13, trisomy 21, absence of *NPM1*, and CN-AML [42]. In sharp contrast with the favorable prognostic effect of gene fusions involving *RUNX1*, *RUNX1* mutations are associated with resistance to standard induction therapy and with inferior overall survival, for both younger and older patients, and are an adverse risk factor in the 2017 ELN guidelines [43].

### CCAAT Enhancer Binding Protein α (*CEBPA*)

*CEBPA* is a transcription factor that plays a key role in hematopoiesis and is a master regulator of myeloid differentiation [44]. Mutations occur in 6 to 10% of AML cases [45] and are identified in both amino and carboxy-terminal regions, with the latter resulting in a truncated protein that is unable to dimerize and bind DNA [46]. Only bi-allelic, not single, *CEBPA* mutations predict an increased CR rate and favorable survival. *CEBPA* mutations can also be inherited through the germline, with this subset of patients often going on to develop AML with the acquisition of additional mutations that include but are not exclusive to the other *CEBPA* allele [47].

## Tumor Suppressor Gene Mutations

### TP53

Mutations in the tumor suppressor *TP53* are identified in 8% of AML cases and are associated with complex karyotype, therapy-related AML, chemo-resistance, high relapse rate, and poor survival [48]. These mutations also confer an adverse risk in the European Leukemia Net (ELN) guidelines and patients that carry a TP53 mutation are poorly served by current therapeutic strategies.

## The Evolution of AML from a Latent Preleukemic State

More recently, AML-associated mutations have been found in healthy aging individuals, a condition named age-related clonal hematopoiesis (ARCH) or clonal hematopoiesis of indeterminate potential (CHIP), and its incidence is as high as 10% in individuals over 65 years of age. These individuals have a higher risk of hematological malignancies and cardiovascular disease [49, 50]. The acknowledgment that AML has a period of latent preleukemia of at least several years in many cases has been a major step forward in understanding the evolution and kinetics of this aggressive disease. Most cases of ARCH involve mutations in epigenetic regulators such as *DNMT3A*, *ASXL1*, and *TET2*, whereas *FLT3* and *NPM1* mutations are never observed, suggesting that these are later cooperating events. Interestingly, despite the fact that ARCH is very prevalent in the general aging population, AML remains a rare disease (~ 4 cases per 100,000 individuals) [51]. Currently, the rate of transformation of ARCH into AML is predicted to be between 0.5 and 1% per year [52]. Deep sequencing of historical samples of patients that have gone on to develop AML has suggested that mutations in *TP53*, *IDH1*, and 2 and RNA splicing factors (*SRSF2*, *SF3B1*, and *U2AF1*) are associated with the highest odds of transformation. In these large cohort studies, *DNMT3A* and *TET2* mutations appeared as commonly occurring events in both AML and control cases, but higher VAFs (> 10%), the presence of a higher number of variants (two or more), along with clonal complexity, correlated with a greater risk of AML [53•, 54•].

The recent identification of a true preleukemic state has changed our view on AML and has identified the possibility of a new treatment paradigm; prevention of the evolution of this deadly disease during its latent phase, with much research, currently devoted to this. The highest risk individuals seem to be those with a detectable TP53 clone [54•], but at the moment, there are no established strategies to eliminate such clones. *IDH* and splicing factor mutant clones have potential therapeutics; however, these treatments are not without toxicity, and a greater predictive capacity, less toxic therapies, and careful clinical trials are needed to justify widespread preemptive intervention.

## Prognostic and Therapeutic Implications of Defining the Molecular Landscape of AML in Individual Patients

The most immediate consequence of the next-generation sequencing revolution has been to improve the risk stratification of AML patients. Indeed, in 2017 the European Leukemia Network recognized the prognostic value of some of the mutations discussed above and updated their risk stratification criteria (Table 1) [55].

**Table 1:** 2017 ELN risk stratification by genetics

Risk category	Genetic abnormality
Favorable	t(8;21)(q22;q22.1); RUNX1-RUNX1T1 inv. (16)(p13.1q22) or t(16;16)(p13.1;q22); CBFB-MYH11 Mutated NPM1 without FLT3-ITD or with FLT3-ITD^low^ Bi-allelic-mutated CEBPA
Intermediate	Mutated NPM1 and FLT3-ITD^high^ Wild-type NPM1 without FLT3-ITD or with FLT3-ITD^low^ (without adverse risk genetic lesions) t(9;11)(p21.3;q23.3); MLLT3-KMT2A Cytogenetic abnormalities not classified as favorable or adverse
Adverse	t(6;9)(p23;q34.1); DEK-NUP214 t(v;11q23.3); KMT2A rearranged t(9;22)(q34.1;q11.2); BCR-ABL1 inv. (3)(q21.3q26.2) or t(3;3)(q21.3;q26.2); GATA2,MECOM(EVI1) -5 or del(5q); −7; −17/abn(17p) Complex karyotype, monosomal karyotype Wild-type NPM1 and FLT3-ITD^high^ Mutated RUNX1 Mutated ASXL1 Mutated TP53

Several studies continue to suggest that incorporating a broader list of gene mutations than indicated by ELN 2017 could further refine risk stratification, but more importantly, it is becoming clear that the prognostic effect of a given mutation depends on the pattern of co-occurring mutations. For instance, Papaemmanuil et al. described how the negative effect of a *FLT3 ITD* in patients with an *NPM1* mutation is much more pronounced when *DNMT3A* is also mutated. Likewise, in patients with an *NPM1* mutation, the presence of a *RAS* mutation improved survival more in the presence than in the absence of a *DNMT3A* mutation. Analogously, the adverse effect of an *MLL* aberration noted in European Leukemia Net (ELN) 2017 depended on the presence of *FLT3 TKD* mutations, and although European Leukemia Net (ELN) 2017 regarded mutations in *IDH2* or in *DNMT3A* as having no prognostic effect, prognosis became considerably worse when both *IDH2* and *DNMT3A* mutations co-occurred in a large series of patients [9••]. However, to take full prognostic advantage of the genetic complexity in AML, where there are usually thought to be between 3 and 5 driver mutations per patient, will require large patient numbers and international collaboration as is planned by large consortia such as the HARMONY alliance (https://www.harmony-alliance.eu).

In addition to the presence of genetic abnormalities at diagnosis, the continued presence of particular gene mutations during or after treatment carries prognostic information for certain genetically defined AML subtypes. In *NPM1*-mutated AML, for instance, detection of mutant *NPM1* transcripts by sensitive quantitative RT-PCR after 2 cycles of chemotherapy had an 86% cumulative incidence of relapse vs 34% for *NPM1* negative patients [56•]. Minimal residual disease (MRD) detection, either by genetics or by multiparameter flow cytometry (MPFC), has therefore assumed a role in risk-adapting post-remission therapy, which had previously been based solely on pretreatment variables. Interest has more recently turned to establishing if a variety of mutations identified through NGS are persistent after treatment and the potential prognostic implications. It is becoming apparent that while detection of ARCH mutations (*DNMT3A*, *TET2*, *ASXL1*) has no prognostic implication and simply underlies the advantage of these clones over wild type in repopulating the marrow after chemotherapy, persistence of non-ARCH mutations is associated with higher cumulative incidence of relapse and shorter survival and relapse-free survival [57], albeit that the resolution of NGS (10^-2^-10^-3^) is more limited than either MPFC or PCR.

**Fig. 1:**
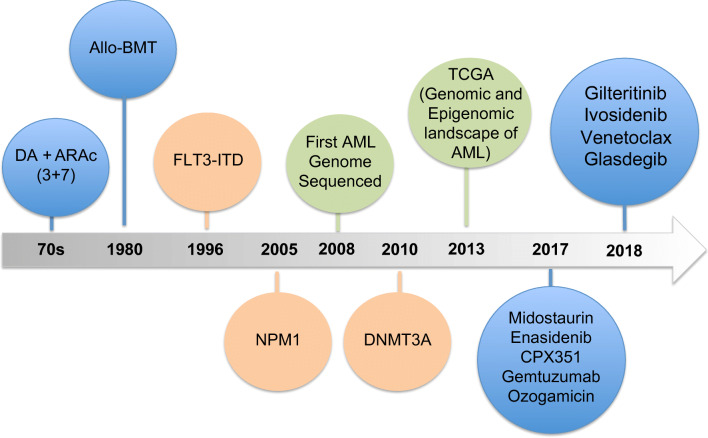
Timing of the identification of mutations associated with AML and evolution of therapeutic strategies. More widespread use of sequencing technologies has enriched the landscape of mutations that are associated with AML. An enhanced ability to diagnose and prognosticate is now translating into an increased understanding of therapeutic vulnerabilities and the development of new therapies. Between 2017 and 2018, the FDA has approved eight novel drugs for the treatment of AML and for the first time in almost 40 years, and AML patients can benefit from a more individualized treatment approach

Finally, the research-based advances described above are translating into clinical practice leading to a very much overdue update in the treatment strategies available to combat AML, and as a result, within the last 2 years, the US Food and Drug Administration (FDA) approved eight novel therapies for patients with AML, many of which are also becoming available in Europe (Fig. 1). Midostaurin, a *FLT3* inhibitor, was one of the first novel therapies to enter clinical practice and is currently recommended in combination with chemotherapy and as a single agent for maintenance therapy in patients with mutated *FLT3* [58]. Similarly, the IDH1 and IDH2 inhibitors ivosidenib and enasidenib have shown promising results in clinical trials and have been approved for use in AML patients with *IDH1* and *IDH2* mutations, respectively [59, 60]. To further champion personalized medicine in AML, the Leukemia and Lymphoma Society is sponsoring a “BEAT AML” trial that plans to recruit 500 patients aged 60 or above with newly diagnosed AML. The trial aims firstly to assess the feasibility of enrolling patients based on mutational status. Following a genomic screening whose results should be available within 7 days of a marrow sample being taken, patients will be assigned to one of several arms based on their genetic profile. The results of this and other similar trials proposed or in set up are eagerly awaited as they will not only pave the way towards the use of precision medicine in AML but also will begin to test the efficacy of several novel targeting agents in a cohort of newly diagnosed older patients, who are the patient group with the highest mortality rates and lowest tolerance for toxic therapies.

## Conclusions

The advent of rapid and affordable genome sequencing has revolutionized our approach to the classification, prediction, and prognostication of acute myeloid leukemia and has greatly improved our scientific understanding of the pathophysiology of this deadly disease. This has initially translated into an improved ability to determine which patients will benefit most from allogeneic stem cell transplantation in first remission, but now, thanks to a growing therapeutic armamentarium, is at last leading to a long overdue clinical progress in AML therapy and to the promise of individualized approaches to improve outcomes in this deadly disease.
